# A retrospective study of ductoscopy combined with immediate methylene blue staining in nipple discharge diseases

**DOI:** 10.1038/s41598-023-46821-6

**Published:** 2023-11-07

**Authors:** Wen-shi Yang, Yan Zhang, Hong-ling Wang, Feng-feng Zhang

**Affiliations:** Department of Breast and Thyroid Surgery, The Central People’s Hospital of Tengzhou, Tengzhou, 277500 China

**Keywords:** Diseases, Medical research

## Abstract

This study investigated the effect of fiberoptic ductoscopy (FDS) combined with methylene blue staining immediately after FDS procedure on pathological nipple discharge diseases. A retrospective analysis of the clinical data of 122 patients with nipple discharge, who underwent FDS and surgical treatment at the Department of Breast and Thyroid Surgery of Tengzhou Central People’s Hospital, was conducted. The demographic characteristics and surgical outcomes of all patients were assessed. According to the injection time of methylene blue, the patients were divided into the control and the observational groups. In the observational group, methylene blue was injected immediately after ductoscopy and then surgical treatment was performed 12–24 h later, while in the control group, methylene blue injection was just few minutes before surgery treatment. There was no significant difference in the demographic characteristics between the two groups such as age and disease course, in the observational group, the incision length 2.39 (0.48) cm, the volume of resected tissue 41.93 (40.57) cm^3^, the intraoperative blood loss 12.19 (2.10) ml and the operation duration 26.95 (4.51) min were significantly lower than those of the traditional group (P < 0.05). The average hospital stay 3.08 (0.62) days, breast shape satisfaction 4.78 (1.63) points and postoperative drainage tube placement [3 (5.08%) days] in the observational group were significantly better than those in the control group (P < 0.05). FDS combined with immediate methylene blue staining, which has the advantages of accurate location of the diseased duct, small surgical incision, less tissue removal, and ease of finding the orifice of discharged mammary duct, and is worthy of widespread clinical application.

## Introduction

Spontaneous nipple discharge (SND) is a common clinical symptom of breast diseases, accounting for 4.8–7.4%^1^. The pathological discharge is commonly due to intraductal lesions^2^. Conventional diagnostic methods such as ultrasound, smear cytology, mammography and galactography, are all indirect diagnostic methods that can neither directly view the lesions in the mammary duct nor accurately locate the lesions. Hence, they are not optimal for the differential diagnosis of pathological nipple discharge^3^. FDS can directly observe the intraductal lesions, allowing for facilitating a targeted surgery^4^. At present, the location of the diseased mammary duct is usually placing a positioning needle under the FDS before the operation or injecting methylene blue during the operation. Disadvantages of the former are as belows: expensive, the risk of dislodging and the inability to locate the lesions of the terminal mammary duct. Traditionally, the operation of intraductal lesions is guided by injection of methylene blue during surgery. However, the extravasation of dye during the operation results in a wide area of unclear blue staining in the operation field,and it is unclear, resulting in extensive tissue stripping and injury during the operation^5^. In this study, for patients with clear surgical indications and the intention to operate, the appropriate amount of methylene blue was injected through the working channel immediately when performing FDS, and the application value of FDS combined with immediate methylene blue staining in pathological nipple discharge was discussed.

## Methods

### Study design and patients

The clinical data of patients with nipple discharge admitted to the breast and thyroid surgery department of Tengzhou Central People’s Hospital between January 2020 and January 2021 was collected. Inclusion criteria were as follows: (1) intraductal lesions requiring surgical biopsy; (2) all patients underwent breast ultrasound, mammography and FDS; (3) the lesion was located in unilateral breast and single orifice of mammary duct; (4) informed consent of patients and their families was obtained; (5) did not receive other treatment before admission and underwent surgery 12–24 h after FDS. Exclusion criteria were as follows: (1) bilateral nipple discharge or porous discharge of unilateral breast; (2) no definite space-occupying lesion; (3) lactating or pregnant women; (4) previous medical history of breast cancer; (5) history of surgical treatment of breast duct disease. The study was conducted in accordance with the Declaration of Helsinki, and the protocol was approved by the Ethics Committee of Tengzhou Central People’s Hospital. All patients signed informed consent forms.

### Ductoscopic examination

The patient was placed in supine position with the affected breast fully exposed. The nipple was considered as the center for routine disinfection and draping, and the surface of the affected nipple was washed with iodophor solution. The offending duct was identified through gentle clockwise pressure on the areola and noticing the source of discharge. The needle of a 1 ml syringe without a tip was inserted from the orifice of the discharging duct, and 1 ml of 1% lidocaine was injected for anesthesia. After catheter dilation with duct dilator from thin to thick, the FDS was inserted into the offending duct, and normal saline was slowly injected to keep the breast tube dilated. During the whole operation, the direction of the FDS was the same as that of the breast tube, from shallow to deep, and the cavity was searched. The breast antrum and all levels of breast ducts were sequentially observed on the display screen, and whether the breast ducts expand under the FDS, presence of flocculent sediment, bloody discharge, and solid space occupation were recorded. After the examination, the iodophor solution was used for disinfection, the operation area was covered with sterile gauze, and the patient was prohibited from bathing within 24 h.

### Duct excision

#### The observational group

For patients with surgical indications such as intraductal lesions lesions in the mammay duct, the lens sheath was fixed, 1.0 ml methylene blue was injected immediately through the working channel of FDS. Surgery was performed 12–24 h after methylene blue staining.

#### The control group

Patients with surgical indications after ductoscopy were admitted to hospital without injection of methylene blue. The breast gland was pressed during the operation, and the sterile needle was inserted from the orifice of the discharging duct to inject 1.0 ml methylene blue.

A drainage tube was placed depending on the extent of the tissue removed during the operation. If the pathological result was breast cancer, modified radical mastectomy or breast-conserving surgery was performed for breast cancer, and relevant postoperative treatment was performed according to the NCCN breast cancer guidelines.

#### Observational indicators

(1) Cut length; (2) volume of tissue removed; (3) intraoperative bleeding volume; (4) operation duration; (5) average hospitalization time; (6) the satisfaction degree of breast shape was evaluated by the Vancouver Scar Assessment Scale^6^. The satisfaction degree was 0–5 points, the average degree was 6–10 points, the dissatisfaction degree was 11–15 points; (7) postoperative drainage tube placement.

### Statistical analysis

The count data was expressed as percentage (%) and compared by χ^2^ test and Fisher’s exact probability method. Continuous data were presented as the mean ± standard deviation (x ± s) and compared by t-test. P < 0.05 was considered statistically significant. Statistical analyses were performed using SPSS 17.0 statistical software (SPSS, Inc., Chicago, IL).

### Ethical approval

This study was conducted in accordance with the principles of the Helsinki Declaration. Approved by the Ethics Committee of Tengzhou Central People’s Hospital (Date: March 20, 2023/NO.2023-Ethical Review-29).

### Consent to participate

The Institutional Review Committee of Tengzhou Central People’s Hospital classifies such studies as retrospective studies and therefore does not require informed consent.

## Results

Four patients with bloody discharge without obvious space-occupying lesions under FDS were diagnosed as malignant tumor by histopathology, and were excluded from the statistical analysis. A total of 118 patients were included in this study.

### Control group results

The mean age of patients was 38.44 ± 7.99 years. The course of disease was ≤ 1 month in 34 cases, 1–3 months in 10 cases, and > 3 months in 15 cases. There were 38 cases of left nipple discharge and 21 cases of right nipple discharge. There were 42 cases of serous discharge, 12 cases of watery discharge, 3 cases of milky discharge, and 2 cases of bloody discharge.

### Observational group results

**Figure 1:**
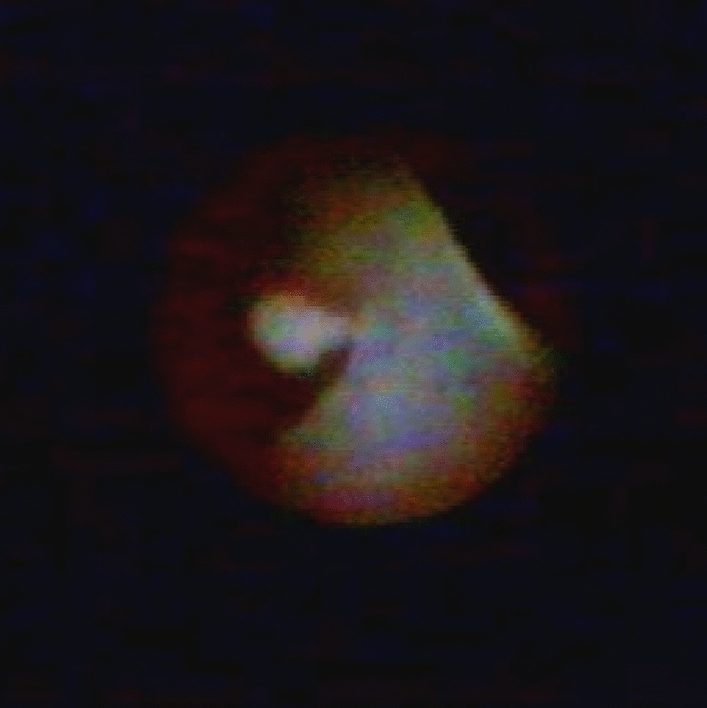
Breast duct occupying lesion with clear color discharge.

**Figure 2:**
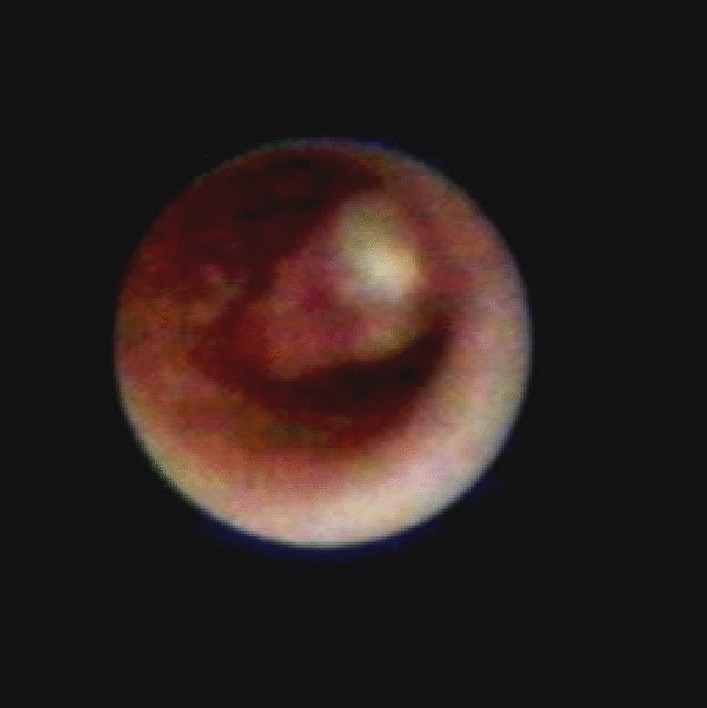
Breast duct occupying lesion with bloody discharge.

**Figure 3:**
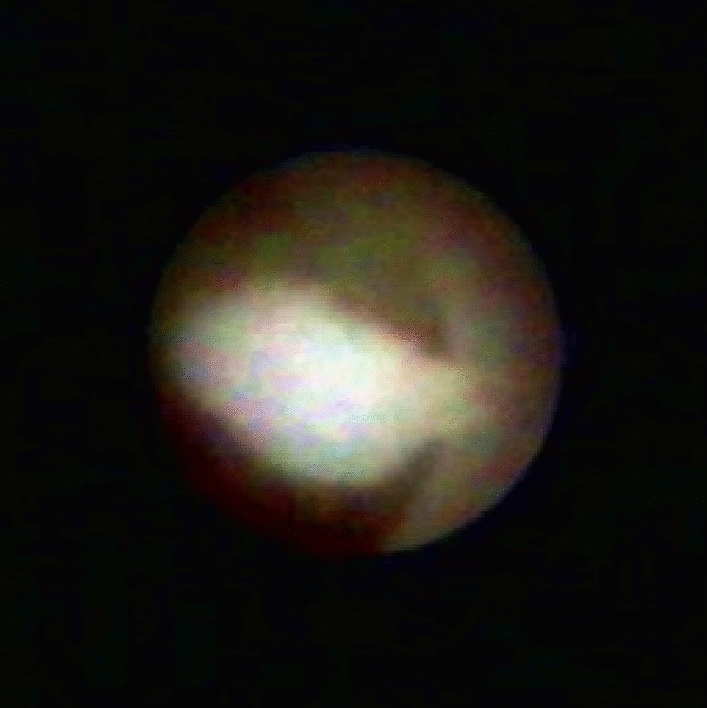
Breast duct occupying lesion with yellow discharge.

**Figure 4:**
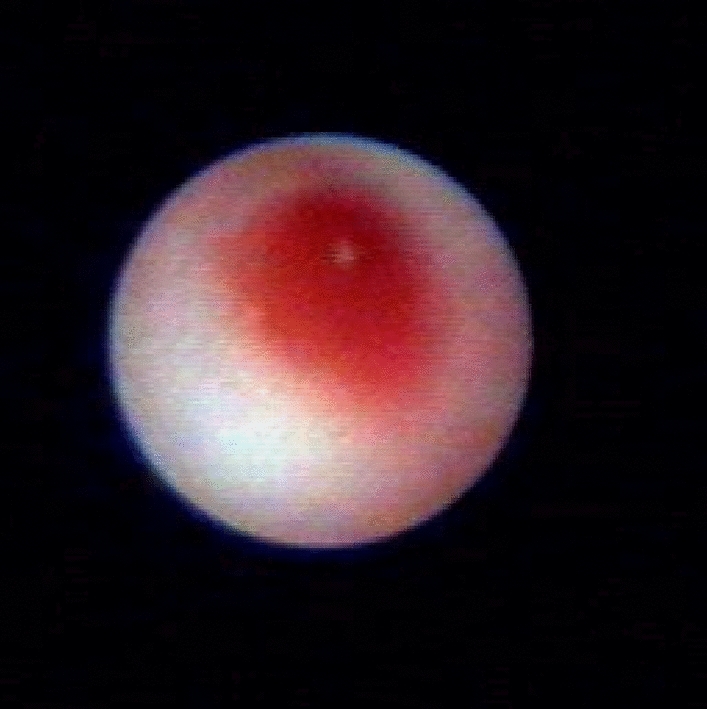
Bloody discharge without space occupying lesion.

The mean age of patients was 41.19 ± 8.18 years, the course of disease was ≤ 1 month in 29 cases, 1–3 months in 12 cases, and > 3 months in 18 cases, there were 42 cases of left nipple discharge and 17 cases of right nipple discharge, there were 39 cases of serous discharge, 16 cases of watery discharge, 2 cases of milky discharge, and 2 cases of bloody discharge. There was no statistically significant difference in the demographic characteristics between the two groups (P > 0.05) (Table 1, Figs. 1, 2, 3, 4).

**Table 1:** Baseline characteristics.

Clinical characteristic	The control group	The observational group	t/x^2^	P
Mean age (SD),years	38.44 (7.99)	41.02 (8.06)	1.75	P = 0.084 > 0.05
Disease course				
≤ 1 month	34	29	0.851	P = 0.653 > 0.05
1–3 months	10	12		
> 3 months	15	18		
Distributions of the lesions				
Left	38	42	0.621	P = 0.431 > 0.05
Right	21	17		
Color of discharge				
Yellow	42	39	1.072	P = 0.83 > 0.05
Clear	12	16		
Milky	3	2		
Bloody	2	2

**Figure 5:**
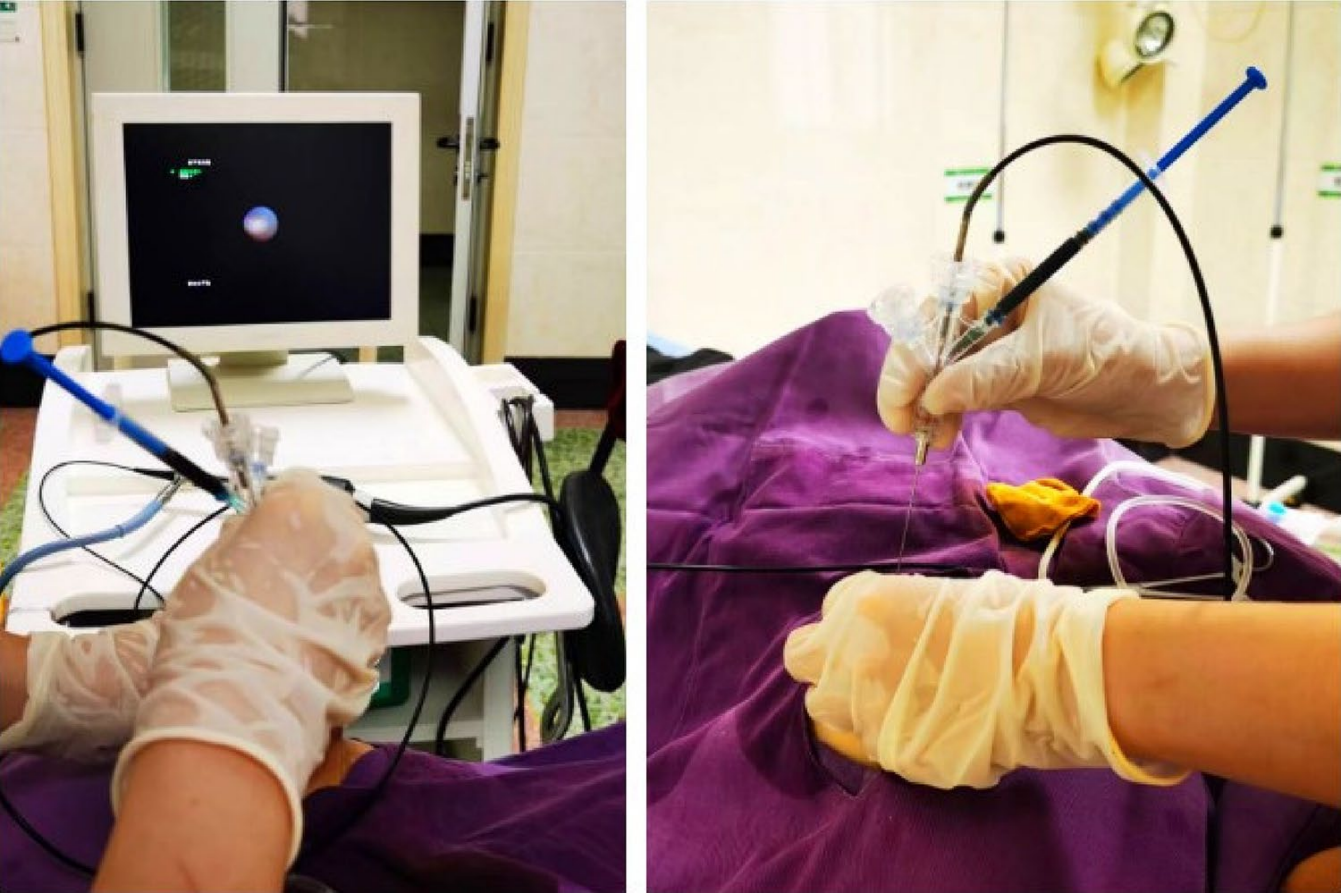
Introduction of the ductoscope.

**Figure 6:**
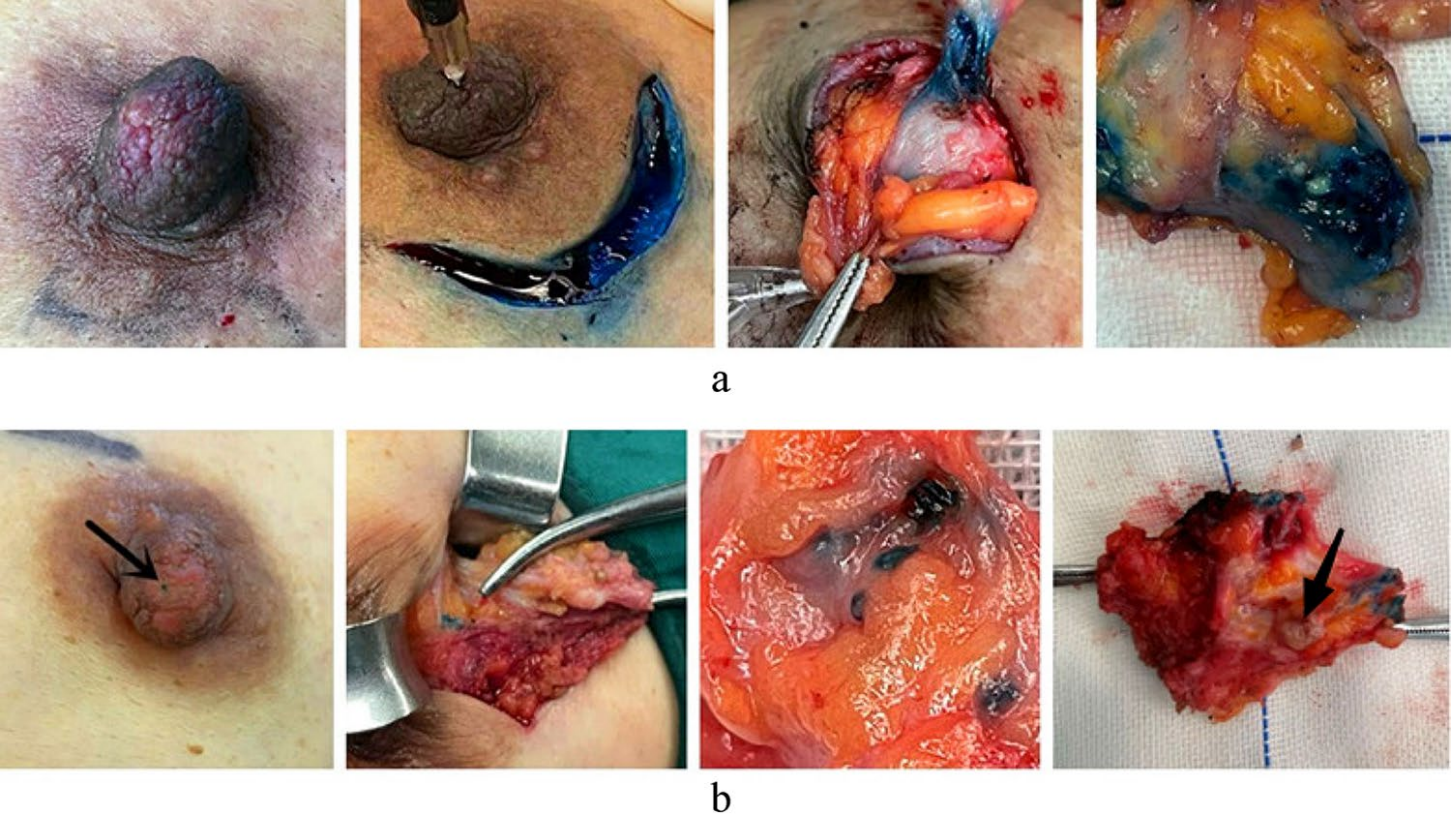
(**a**) Operation of patients with nipple discharge in the traditional group. (**b**) Operation of patients with nipple discharge in the observation group (→ blue dye discharge breast duct opening and intraductal lesion tumor body shown).

In the observational group (Fig. 5), the length of surgical incision, the volume of resected tissue, the volume of bleeding and the duration of surgery were significantly lower than those in the control group (P < 0.05) (Table 2). The average hospital stay, the satisfaction of breast shape and the placement of drainage tube were significantly better than those in the control group (P < 0.05) (Table 3, Fig. 6).

**Table 2:** Comparison of intraoperative indexes between the two groups.

	The control group	The observational group	t	P
Length of surgical incision Mean length (SD), cm	3.14 (0.57)	2.39 (0.48)	7.66	P < 0.05
Volume of resected tissue Mean volume (SD), cm^3^	61.23 (40.37)	41.93 (40.57)	2.59	P < 0.05
Volume of bleeding Mean volume (SD), cm^3^	18.05 (5.88)	12.19 (2.10)	7.22	P < 0.05
Duration of surgery Mean duration (SD), min	38.90 (5.60)	26.95 (4.51)	12.76	P < 0.05

**Table 3:** Comparison of postoperative indices between the two groups.

	The control group	The observational group	t/x^2^	P
Hospital stay Mean day (SD), days	3.98 (0.68)	3.08 (0.62)	7.47	P < 0.05
Breast shape Mean point (SD), points	7.68 (1.34)	4.78 (1.63)	10.54	P < 0.05
Drainage tube N (% )	12 (20.34)	3 (5.08)	6.19	P < 0.05

One patient in the observational group with serous discharge diagnosed as intraductal papilloma during her first hospitalization, was finally diagnosed as breast malignant tumor by histopathology and then admitted to our hospital for further treatment again. 2 patients with bloody discharge in the control group were diagnosed as intraductal papilloma, and another 2 patients with bloody discharge in the observational group were diagnosed as intraductal papilloma and atypical hyperplasia. 3 patients in the control group diagnosed as intraductal papilloma under FDS, were finally diagnosed as breast adenosis with atypical hyperplasia of ductal epithelium, with the diagnostic accuracy of 94.9% (56/59). Five patients in the observational group diagnosed as intraductal papilloma under FDS, were finally diagnosed as breast adenosis with atypical hyperplasia of ductal epithelium, with the diagnostic accuracy of 89.83% (53/59). There was no statistically significant difference between the two groups (P = 0.490 > 0.05) with the Fisher’s exact test (Table 4).

**Table 4:** FDS results (based on histopathology).

FDS^a^ diagnosis	Histopathology			Diagnostic accuracy (%)	P^b^
	Intraductal papilloma	Atypical hyperplasia	Malignant tumor		
The control group					0.49
Intraductal papilloma	56	3	0	94.9% (56/59)	
Malignant tumor	0	0	0		
The observational group					
Intraductal papilloma	53	5	1	89.83% (53/59)	
Malignant tumor	0	0	0		

^a^Fiberoptic ductoscopy.

^b^Fisher’s exact test.

## Discussion

Nipple discharge is the third major symptom of breast diseases, after breast lump and pain^7^, accounting for about 7% of breast surgeries^8^. Nipple discharge is divided into physiological discharge and pathological discharge. Pathological nipple discharge (PND) is considered as spontaneous, persistent, emanating from a single duct, and watery, serous, or bloody. The main course of PND is benign, of which intraductal papilloma is account for 40%.However, malignancy is still up to 20%. In our study, benign lesions accounted for 95.90% (117/122) of PND, and malignant tumors accounted for 4.10% (5/122).

Since 1991, Okazaki et al. got a direct view of the breast ducts with FDS, it has been more and more used widely in clinical practice. Once a intraductal lesion was found by ductoscopy, pathology of the lesion is essential. At present, microdochectomy and major duct excision are still utilized for pathology. The lesions inside the mammary duct are usually too small to find. Therefore, accurate positioning of intraductal lesions is particularly important. There are many reports about the direct-view and methylene blue staining of breast ductoscopy^9,10^, but there are few reports discussing and comparing the positioning methods. Ductoscopy wire has the disadvantages of the detachment, displacement and high cost of the positioning wire^11^, which only locate a single lesion found in the mammary duct.

Methylene blue is an effective and cheap dye, and clinicians have mastered its application in breast disease localization. In this study, the control group of patients with PND underwent FDS in the outpatient department, and those with clear indications for surgery were admitted to the hospital. A few minutes after the injection of methylene blue into the orifice of the discharging duct, surgery was then performed. According to our study, we found that the overflow of the mammary duct disappeared after the examination of ductoscopy in some patients, resulting in the inability to accurately locate the orifice of the duct during the operation. On the contrary, the discharging duct of the patients in the observational group were immediately injected with methylene blue through the working channel at the end of ductoscopy, surgery was performed 12–24 h later. During the operation, obvious blue staining was observed on the surface of the nipple (Fig. 6b), which effectively solved the problem of finding the orifice of the mammary duct blindly.

The results of this study showed that the length of surgical incision, the amount of tissue removed, the blood loss and the duration of surgery in the observational group were significantly lower than those in the control group (P < 0.05). The main reason was that some patients in the control group had no overflow after the examination of ductoscopy, so the surgeons blindly chose to inject methylene blue into the orifice of the mammary duct during the operation, which is easy to penetrate the mammary duct and form a false channel, resulting in inaccurate positioning. Methylene blue injected during the operation can easily seep into the surrounding tissues outside the mammary duct due to high pressure inside the duct or improper technique of the operator. Methylene blue can easily diffuse and metabolize rapidly in breast tissue^11^, resulting in a wide area of blue staining, thus blurring the field of vision. It is necessary to further extend the incision to remove a wider area of blue-stained glands, resulting in increased intraoperative blood loss, large amount of resected tissue, and extension of the operation time. The observational group was significantly better than the control group in terms of the average postoperative hospital stay, the satisfaction of breast shape and whether the drainage tube was placed or not (P < 0.05). The potential reasons included extravasation of methylene blue, excision of blue-stained tissues, unclear visual field, etc., which were closely related to the large amount and area of resected tissue.

The dyeing area and dyeing intensity of methylene blue decrease with the dyeing time^11^. When the dyeing time is over 16 to 20 h, the residual staining disappears linearly, punctately or completely. In this study, patients in the observational group received surgical treatment 12–24 h after the injection of methylene blue during the process of ductoscopy. Compared with the patients in the control group, methylene blue that leaked from the duct into the surrounding tissue was absorbed after 12–24 h, which inside the duct still persisted. Only the blue stained ducts and a small amount of surrounding breast tissue were removed, thus avoiding excessive damage to the breast. In this study, the volume of resected tissue in the observational group 41.93 (40.57) cm^3^ was significantly smaller than that in the control group 61.23 (40.37) cm^3^. In addition, the less breast tissue is removed, the less damage it will cause to the breast’s lactation function of the childbearing-age women^12^. Moreover, on the excised specimen, the blue stained mammary ducts were visible clearly, and the intraductal lesions can be more easier found along the blue stained ducts by surgeons or pathologists, even the small lesions in the terminal ducts were not easily missed. The cost of the FDS and methylene blue are less, which reduces the economic burden of patients, saves medical resources, and improves patients’ satisfaction.

In this study, four patients had bloody discharge, and no clear space occupying lesion in the duct was found by ductoscopy, yet they took active surgical treatment, and the final postoperative pathological diagnosis was breast cancer. According to the relevant literature^13^, most of the breast malignant lesions are concentrated in the terminal breast duct, which are beyond the scope of FDS and not easily diagnosed. Some lesions are not protrued into the ducts, but only invade the breast duct wall to cause capillary rupture and bleeding, resulting in bloody discharge. Therefore, we believe that for patients with bloody nipple discharge, active treatment should be undertaken to avoid missed diagnosis of breast cancer.

This study had certain limitations. When there is no active discharge, FDS cannot be performed, because it’s difficult to insert FDS into a normal mammary duct. Because the mammary duct is very thin, sometimes fibrous tissue or inflammatory secretions inside the duct can block the field of view, resulting in a lack of clear vision under ductoscopy, meanwhile lesions located in the terminal ducts may be missed because of the limitations of the ductoscopy’s length and width. So we recommend that patients with PND undergo combined examinations such as ultrasound, MRI, etc.when necessary. There is no consensus on the time interval between the injection of methylene blue and the surgical treatment. Therefore, subgroup analysis of the time between the injection of methylene blue and surgery should be performed to provide surgeons with more accurate operation time.

## Conclusion

FDS combined with immediate methylene blue staining is an easy mastered method for surgeons, which can accurately locate the discharging ducts and the intraductal lesions, provide clear vision of surgical field, reduce the amount of dissected tissue and improve patients’ satisfaction. It has significant clinical effects, and is worthy of extensive promotion in the diagnosis and surgical treatment of patients with PND.

## Data Availability

The datasets generated during and/or analysed during the current study are not publicly available due to the data in this study involve patient privacy issues, and there are follow-up studies that are still ongoing, and the data are confidential for the time being but are available from the corresponding author on reasonable request.
